# Importance of Dietary Changes During the Coronavirus Pandemic: How to Upgrade Your Immune Response

**DOI:** 10.3389/fpubh.2020.00476

**Published:** 2020-08-27

**Authors:** Ali Chaari, Ghizlane Bendriss, Dalia Zakaria, Clare McVeigh

**Affiliations:** Premedical Department, Weill Cornell Medicine, Qatar Foundation, Education City, Doha, Qatar

**Keywords:** COVID-19, coronavirus, immune system, balanced diet, micronutrients, macronutrients, probiotics, intermittent fasting

## Abstract

The new coronavirus pandemic continues to spread causing further public health, social, and economic issues. The disparities in the rates of death between countries poses questions about the importance of lifestyle habits and the immune status of populations. An exploration of dietary habits and COVID-19-related death might unravel associations between these two variables. Indeed, while both nutritional excess and deficiency are associated with immunodeficiency, adequate nutrition leading to an optimally functioning immune system may be associated with better outcomes with regards to preventing infection and complications of COVID-19, as well as developing a better immune response to other pathogenic viruses and microorganisms. This article outlines the key functions of the immune system and how macronutrients, micronutrients, and metabolites from the gut microbiome can be essential in the development of an efficient immune system. In addition, the effects of intermittent fasting on the inflammatory state as well as metabolic parameters will be discussed.

**Graphical Abstract:**
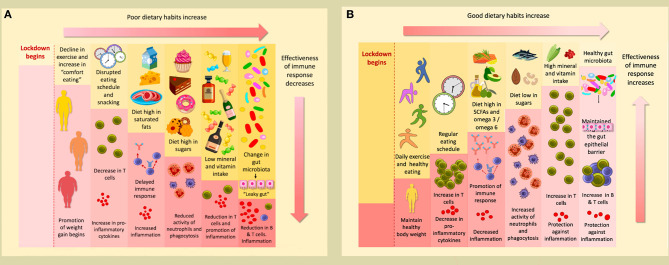
**(A)** The conditions of lockdown tend to promote poor dietary habits; a decline in exercise and increase in comfort eating promotes the weight gain that many have experienced during this time. A disrupted eating schedule and frequent snacking can result in a decrease in T cells and an increase in pro-inflammatory cytokines. A delayed immune response and increased inflammation can occur as a consequence of a diet high in saturated fats. Furthermore, a high sugar diet reduces the activity of neutrophils ad phagocytes. This will be accompanied by a reduction in T cells and promotion of inflammation if levels of minerals and vitamins are insufficient. Finally, poor dietary habits change the gut microbiota, causing “leaky gut,” which results in a reduction of B & T cells, and subsequent inflammation. **(B)** Conversely, following good dietary habits and undertaking daily exercise during lockdown helps maintain a healthy weight. If a regular eating schedule is followed, with well-spaced meals, levels of T cells will actually increase, and there will be a reduction in pro-inflammatory cytokines. A diet rich in SCFAs and with a high omega 3/omega 6 ratio will promote a strong immune response and decrease inflammation. If sugars are consumed only at low levels, the activity of neutrophils and phagocytes will increase. An accompanying increase in T cells will occur if an adequate level of minerals and vitamins are consumed; they will also protect against inflammation. These affects will also be promoted if a healthy gut microbiome is maintained to preserve the integrity of the gut epithelial barrier.

## Introduction

In the past two decades, the world has seen the emergence of three novel coronaviruses (CoV) leading to disease outbreaks that have caused considerable global health consternation: the severe acute respiratory syndrome coronavirus (SARS-CoV), the Middle East respiratory syndrome coronavirus (MERS-CoV), and the recently emerged coronavirus SARS-CoV-2 (1–3).

COVID-19 is the name of a newly identified disease caused by SARS-CoV-2, and it was originally observed as a cluster of atypical pneumonia cases occurring in Wuhan, China, in December 2019 (2). While this newly identified virus belongs to the same β-coronavirus genus as SARS-CoV and MERS-CoV, the novel disease seems to be characterized not only by mild upper respiratory infections, similar to other corona-viruses, but also by the presence of symptoms of the lower respiratory tract that are sometimes very severe (4). These mild and even asymptomatic cases have contributed to the silent spread of infections worldwide, increasing the probability of infecting high risk groups of individuals comprising immunocompromised patients and those with chronic diseases (1, 4–12). Indeed, the WHO has estimated the reproductive number (R_0_) of the novel infection by SARS-CoV-2 to range between 2 and 2.5, which is higher than SARS (1.7–1.9) and MERS (<1), suggesting from the outset that COVID-19 has a higher pandemic potential (9, 10).

Some studies have shown that patients with COVID-19 experience a dysregulation of their immune response (13). Conversely, other studies have emphasized how some individuals can recover from COVID-19 symptoms within days; an effective immune response was found to be associated with successful clinical recovery (14). Many studies have highlighted the important role of the human innate and adaptive system in COVID-19 pathophysiology (15, 16). Furthermore, there is evidence that environmental factors, such as unbalanced nutrition, toxins, and inflammation, and the sudden lifestyle changes that occur during quarantine/lockdown can cause physicochemical and psychological stress. These factors may lead to a compromised immune system and deregulate the immune system, making the human body more vulnerable to viral infections (17–20).

An optimal nutritional state has been found to be essential for a well-functioning immune system and for the protection against viral infections (21). Furthermore, malnutrition and/or an unbalanced diet represent an important cause of immunodeficiency worldwide, with infants, children, adolescents, and the elderly being the most affected (22, 23). In this context, deficiencies in essential nutrients are associated with an impairment of cell-mediated immunity, phagocyte function, complement system, and cytokine production in humans (22, 23). Moreover, deficiency in micronutrients such as vitamins, minerals, and polyphenols has been shown to have profound consequences for immune system functioning and susceptibility to infection. Carotenoids, vitamins, selenium, zinc, and polyphenols, as well as many other nutrients, have been shown to modulate the immune system. Furthermore, dietary manipulation of these micronutrients has been shown to alter immune function (21, 24–26). Nutritional excess of carbohydrates, saturated fats, coupled with physical inactivity leading to obesity, can also deregulate the immune system of the host thereby increasing susceptibility to infection (21, 27). Unfortunately, the quarantine and self-isolation of many individuals during the current pandemic promotes these unhealthy behaviors.

This narrative review principally aims at revealing the benefits of balanced nutrition in prevention and treatment of viral infection, by strengthening the immune system. We will be discussing benefits of a number of macro and micronutrients as well as their mechanisms of action.

In addition, evidence is emerging that chronic diseases are strongly associated with the severity of the symptoms and prognosis (11) but the mechanisms explaining this relationship are still unclear and are being explored. Unfortunately, only a limited amount of clinical data is available to draw direct conclusions on the potential of nutritional changes in protecting individuals from COVID19. However, we believe that it is important to note that any changes in lifestyle can also greatly impact chronic diseases in non-infected chronic patients with a high risk for severe COVID-19 disease and thereby *indirectly* affect their response to SARS-CoV-2 infections. Therefore, information regarding the effects of nutritional changes, including fasting, in reversing dysbiosis and chronic diseases in non-infected High Risk for Severe Illness (HRSI) individuals is also included in this paper.

## Human Coronavirus Infection and the Host's Immune System

### Components of the Human Immune System and Lines of Defense Against Viral Infection

The first line of immune defenses includes the physical and chemical barriers that attempt to block the entry of microbes. When these barriers are breached, the microbes will be fought by the components of the internal innate immune system which is composed of leukocytes and defensive proteins that act immediately and non-specifically to eradicate infections (28). If innate immunity fails to eliminate the infection, the adaptive immune system will be activated. T and B lymphocytes are the adaptive immune cells which are able to recognize antigens with high specificity (28, 29). Table 1 summarizes the major functions of the innate and adaptive immune cells.

**Table 1:** Summary of the major functions of the innate and adaptive immune cells.

**Innate leukocytes**	**Description and function**	**References**
Mast cells	Produce/secrete proinflammatory mediators such as cytokines, eicosanoids, and vasoactive amines such as histamine, which causes vasodilation and increases vascular permeability.	(30)
Macrophages	Phagocytes that ingest and destroy microbes. They also produce inflammatory cytokines.	(31)
Monocytes	Circulating phagocytes which can ingest microbes in blood. They migrate to tissues under inflammatory conditions and differentiate to macrophages. They also produce inflammatory cytokines.	(31)
Neutrophils	Circulating phagocytes/granulocytes. They migrate to tissues under inflammatory conditions and destroy microbes by phagocytosis and degranulation. They also produce inflammatory mediators.	(31)
Eosinophils	Circulating granulocytes. They migrate to tissues under inflammatory conditions and kill parasites.	(32)
Basophils	Circulating granulocytes. They migrate to tissues under inflammatory conditions and kill parasites.	(32)
Natural Killer (NK) cells	They are responsible for killing host cells that are infected, stressed, or damaged. Therefore, they play an important role in the eradication of intracellular pathogens and tumor cells. They also produce inflammatory cytokines.	(33, 34)
Dendritic cells (DC)	They function as antigen presenting cells (APC) which mediate the transition from innate to adaptive immunity. If the innate immune system fails to eliminate infection, DC capture and process protein antigens and present them to T lymphocytes. They produce inflammatory cytokines.	(28, 29)
**Adaptive leukocytes (lymphocytes)**	**Function**	**References**
CD4+ T cells	Upon activation by APC, they become helper T cells (Th1, Th2, or Th17). Some CD4+ T cells are regulatory (Treg).	(28, 29)
	Th1: Activate the M1 pathway of macrophages which induce inflammation. They also produce inflammatory cytokines.	(35)
	Th2: Activate the M2 pathway of macrophages which suppress inflammation.	(36)
	Th17: Produce IL-17 which activates and recruits inflammatory leukocytes to various tissues.	(37)
	Treg: Regulatory CD4+ T cells which have immunosuppressive effect.	(38)
CD8+ T cells	Upon activation by APC, they become cytotoxic T cells (CTL) which are responsible for killing infected, stressed, or damaged host cells.	(28, 29)
B cells	When activated, they produce antibodies that neutralize pathogens and enhance the effector mechanisms of other immune cells such as phagocytes.	(39, 40)

The immune response is triggered by the interaction between the pattern recognition receptors (PRRs) of the host cells and the pathogen associated molecular patterns (PAMPs) (41). The antiviral defense is initiated when PRRs such as Toll-like receptors (TLRs), retinoic acid-inducible gene I (RIG-I)-like receptors (RLRs) or NOD-like receptors (NLRs) bind to viral PAMPs such as DNA, RNA, or proteins (42). This interaction induces some signaling cascades through the activation of different families of transcription factors (43, 44). Type I and Type II interferons (IFN-I and IFN-II) are cytokines produced in response to viral infections (45). IFN-I (IFN-α and β) are produced by various types of cells and interfere with viral replication which creates an antiviral state through various mechanisms (46–48). In addition to directly inhibiting viral replication, IFN-I can modulate the innate and adaptive immunity including the activation of the cytotoxic activity of natural killer (NK) cells and cytotoxic CD8+ T lymphocytes (CD8+ CTL) cells which are essential to eradicate the virally infected host cells. Furthermore, IFN-I can stimulate the production of IFN-γ (IFN-II) by NK cells (49). IFN-γ promotes the macrophages classical pathway (M1) which induces inflammation and promotes the intracellular killing mechanisms. Furthermore, IFN-γ stimulates the differentiation of CD4+ T helper (Th) lymphocytes into Th1 which themselves are major producers of IFN-γ (35). Conversely, Th2 activate the alternative pathway of macrophages (M2) which suppresses inflammation and promotes the repair mechanisms (36). Therefore, the Th1 response, together with the cytotoxic activities of NK and CD8+ CTL, are vital antiviral mechanisms (28, 50).

The inflammasome is an important structure in the antiviral defense which is assembled when cytosolic viral molecules bind to NLR. It induces the activation and secretion of interleukin (IL) 1β which is a potent pro-inflammatory cytokine. Moreover, it induces pyroptosis leading to the host cell death and consequently the control of viral infection (51). Tumor necrosis factor-α (TNF-α) is another potent pro-inflammatory cytokine that can cause host cell apoptosis (52). Both TNF-α and IL-1β induce the expression of adhesion molecules by endothelial cells which is essential for the migration of leukocytes across capillaries as part of the inflammation cascade (52, 53). Inflammation could also be induced by a wide range of cytokines such as IL-6, which, in addition to its pro-inflammatory function, together with transforming growth factor (TGF)-β, stimulate the differentiation of “CD4+ Th cells or Th cells” into the proinflammatory Th17 subset (54, 55). Th17 cells are characterized by the production of IL-17 which plays an essential role in the antiviral defense by activating and recruiting inflammatory leukocytes in various tissues (37). Furthermore, IL-17 was reported to promote an effective Th1 and CD8+ CTL responses in addition to the enhancement of humoral immunity by promoting B cell proliferation and differentiation into plasma cells during viral infections (37, 56). Humoral immunity is an essential arm of the antiviral defenses, providing the antibodies that neutralize the virus and enhancing the effector mechanisms of other immune cells such as phagocytes (39, 40). IL-17 could be also produced by a wide range of immune cells such as NK and γδ T cells (57–59). γδ T cells are a subgroup of T cells that have a different structure of T cell receptors compared with conventional T cells (αβ T cells) which can bind to non-peptide antigens. It has been shown that γδ T cells link innate and adaptive immunity and work as antigen presenting cells (APC) to activate CD4+ Th and CD8+ CTL in addition to their capacity to produce cytokines and lytic enzymes which take part in controlling viral infections (60).

Another type of pro-inflammatory cytokines is the chemokines that induce inflammation by functioning as leukocytes chemoattractants. Examples of chemokines that take part in antiviral defense are monocyte chemoattractant protein-1 (MCP-1), macrophage inflammatory protein-1 alpha (MIP-1), IFN-γ inducible protein (IP-10) and IL-8 which are summarized in Table 2.

**Table 2:** Summary of the major functions of cytokines and chemokines.

**Cytokine**	**Function in antiviral immune response**	**Mechanism of action**	**References**
IFN-I (IFN-α and β)	Antiviral	Interfere with viral replication, activate NK cells, and induce the production of IFN-γ.	(49)
IFN-II (IFN-γ)	Pro-inflammatory	Activates the M1 pathway and promote Th differentiation to Th1.	(35)
IL-1β	Pro-inflammatory	Induces the expression of adhesion molecules by endothelial cells and induce pyroptosis.	(51, 52)
TNF-α	Pro-inflammatory	Induces the expression of adhesion molecules by endothelial cells and induce apoptosis.	(53)
IL-6	Pro-inflammatory	Promotes Th differentiation to Th17 and induce the production of CRP which is part of the acute phase inflammatory response.	(54, 55)
IL-17	Pro-inflammatory	Recruits inflammatory leukocytes to the site of infection, promote an effective Th1 and CD8+ CTL responses and enhance humoral immunity.	(37, 56)
MCP-1	Pro-inflammatory/chemoattractant	Recruits monocytes from blood stream to the site of infection.	(61)
MIP-1α	Pro-inflammatory/chemoattractant	Recruits inflammatory leukocytes to the site of infection.	(62)
IP-10	Pro-inflammatory/chemoattractant	Recruits inflammatory leukocytes and enhance inflammation by promoting the Th1 response.	(63, 64)
IL-8	Pro-inflammatory/chemoattractant	Recruits neutrophils to the site of infection which enhances inflammation.	(65)
G-CSF	Pro-inflammatory	Enhances the production of neutrophils and macrophages and enhances phagocytosis.	(66, 67)
IL-7	Pro-inflammatory	Promotes the development, proliferation, and survival of lymphocytes and suppress the expression of inhibitory molecules by T cells.	(68)
IL-2	Pro-inflammatory/Anti-inflammatory	Enhances proliferation and survival of Th1, Th2, Th17, and Treg.	(69, 70)
IL-4	Anti-inflammatory	Activates the M2 pathway and promote Th differentiation to Th2.	(71)
IL-10	Anti-inflammatory	Regulates inflammation.	(72)

Additionally, some cytokines are required for the development, proliferation, differentiation, and survival of leukocytes and may therefore act as pro- or anti-inflammatory cytokines. For example, granulocyte colony-stimulating factor (G-CSF) enhances the production and function of neutrophils and macrophages and consequently could function as a pro-inflammatory cytokine (66, 67). On the other hand, both IL-7 and IL-2 play a pivotal role in the development and homeostasis of lymphocytes and may induce inflammation (68, 69). However, IL-2 is also required for the development and function of regulatory T cells (Treg) (70). Accordingly, IL-2 may have a dual function as pro-inflammatory or anti-inflammatory cytokine (38, 69). Inflammation could be suppressed by the anti-inflammatory cytokines which are summarized in Table 2.

Despite the vital defensive role of inflammation as a major immune response, it is important to note that in several viral infections, the tissue damage is not directly caused by the virus, it is instead the result of an exuberant inflammatory response to the viral infection (73, 74).

### Human Immune Responses to SARS-CoV-2 Infection

MERS-CoV, SARS-CoV, and SARS-CoV-2 are β-coronaviruses that can cause fatal respiratory tract infections and extra-pulmonary manifestations (75–77). SARS-CoV-2 binds to the angiotensin-converting enzyme 2 (ACE2), which it uses as a receptor to enter the cell (78, 79). ACE2 proteins, part of the renin- angiotensin system (RAS), are found at several locations, including the olfactory epithelium and the gut and are numerous throughout the respiratory epithelial tissue of the lung, kidney, intestine, and blood vessels (80). This may be the cause behind the high incidence of bronchitis and pneumonia in severe COVID-19 infected patients. It has been shown that ACE2 is responsible for the degradation of Angiotensin II resulting in the formation of Angiotensin 1-7, thereby, negatively regulating RAS (81, 82). Besides the role of ACE2 to serve as a functional receptor for SARS-CoV-2, it has been shown that ACE2 is implicated in many pathologies including diabetes, cardiovascular diseases (CVD), and lung diseases (82–84). SARS-CoV-2 appears to use different amino acids in its spike protein for binding the ACE2 receptor with more affinity than previous SARS viruses (85, 86). Interestingly, the latest studies have shown that, after infection, some cellular processes downregulate ACE2 expression (87). Destruction of ACE2 further increases the activity of angiotensin II, which has pro-inflammatory, pro-oxidative, vasoconstrictive, and pro-thrombotic effects that can lead to the thrombotic changes and organ failure that were noted in COVID19 patients and which contributed to death (88). In fact, it seems that after viral infection, ACE2 could play a key protective role in the progression of the disease and the severity of the respiratory distress syndrome (89). A study by Imai et al. (89) published in Nature have shown that ACE2 protects mice form severe acute lung injury after sepsis. Sepsis is characterized by oxidative stress, systemic inflammation, and organ failure that is due to excessive free radical production.

Based on the previous studies conducted on SARS-CoV and MERS-CoV, it could be predicted that the innate immune response against SARS-CoV-2 may start when the viral molecules are recognized by TLRs, RLR, or NLR. This interaction triggers the inflammatory response and stimulates the production of IFN-I which controls viral replication (77). However, it was also reported that SARS-CoV and MERS-CoV may evade the innate immune response by interfering with the IFN-I signaling pathways through various mechanisms. Failure to initiate or complete the IFN signaling cascades during the early phase of infection may result in an uncontrolled viral replication. This may lead to the recruitment of neutrophils and monocytes/macrophages to the infected tissues which results in the excessive production of pro-inflammatory cytokines (90). Accordingly, it could be hypothesized that the exaggerated damaging inflammatory response observed in COVID-19 patients is at least partially attributed to the suppressed/delayed IFN-I pathways accomplished by SARS-CoV-2. Furthermore, in severe COVID-19 cases there is a diminished response of Th1 cells (13).

Several studies have documented that levels of cytokines and chemokines vary according to disease stage and severity of COVID-19. For example, one study showed that plasma levels of IL-2, IL-6, IL-8, IL-10, and TNF-α, were found to be higher in patients with severe infection than those with mild to moderate infection (13). Another study showed a similar trend, with plasma concentrations of IL-2, IL-7, IL-17, IL-10, MCP-1, MIP-1A, IP10, and TNF-α being observed to be higher in COVID-19 patients undergoing treatment in intensive care units than in any other category of COVID-19 patients (91).

In one report, analyzing 99 cases in Wuhan, Zhou and colleagues observed an increase in the total neutrophils (38%), an increase in serum IL-6 (52%), an increase in C-reactive protein (CRP) (84%), and a decrease in total lymphocytes (35%) (92). In another report from Wuhan, analyzing 41 patients, an increase in the total neutrophils and a decrease in the total lymphocytes has been shown, which also correlate with disease severity and death (91). Furthermore, the decreased level of lymphocytes observed by (90), could be explained by the ability of SARS-CoV-2 to infect T lymphocytes, which leads to apoptosis of lymphocytes and consecutive lymphocytopenia (4, 93). In fact, it was found that the absolute count levels of CD4+ and CD8+ T cells were significantly lower in subjects with a severe SARS-CoV-2 infection (94–96). In addition to T cells, the reduction of B cells and NK cells are seen in COVID-19 (13, 97). Therefore, the reduced adaptive immune response against the virus, manifested by an impaired T-cell function, may contribute to the uncontrolled secretion of the pro-inflammatory cytokines in what is known as a “cytokine storm” accompanied with a multi-organ failure (8, 98). Interestingly, one study illustrated how an otherwise healthy individual with a robust immune system is capable of achieving an efficient clearance of SARS-CoV-2, accompanied by clinical recovery after 13 days and full recovery at day 20 after infection (14).

The impact of comorbidity is yet another factor that may affect the outcome of COVID-19. It has been reported that factors such as obesity, diabetes and CVD may increase the risk of progression and mortality among COVID-19 patients (99). One factor that may link such diseases to the increased severity and progression of COVID-19 is inflammation. For example, obesity is associated with metabolic alterations which may dysregulate the immune response through various mechanisms (100). Furthermore, obesity was found to be associated with the increased production of IL-6, TNF-∞, MCP-1, and CRP leading to chronic and low-grade inflammation which may result in defective innate immunity and cause the development of type 2 diabetes and CVD (100, 101). Likewise, the association between diabetes, CVD, and chronic inflammation has been well-established (102, 103). Additionally, studies have shown that ACE2 expression is significantly increased in obese individuals, as the RAS upregulates ACE2 to protect the heart. However, because of this increased ACE2 expression, obese individuals are thought to be more exposed to the SARS-CoV2 viral spread into the lungs. Treatment and close management of obesity is an important approach that needs to be considered to prevent patients from being infected and developing complications.

Therefore, it could be elucidated that the efficiency of the immune response, which is controlled by multiple factors including nutrition, may dictate the outcome of COVID-19. The following section presents a review of the nutritional components that were shown to boost the immune system, including, but not limited to viral infections and coronaviruses.

## The Role of Nutrition in Immune Function

A balanced, adequate diet is required for the cells of the immune system in order to function optimally. During situations with increased requirements (e.g., infection, stress, and pollution), the immune system is activated and thus increases the demand for energy. A balanced, optimal diet strengthens the immune response and supports the function of the immune cells not only by producing an effective response against pathogens, but also by resolving infections in a short time thus avoiding any further chronic inflammation (104). Various nutrients are involved in this process. This section highlights some that have been shown to play specific roles in the development and maintenance of an effective immune system.

### Role of Macronutrients in the Immune Function

#### Effect of Dietary Fats in the Immune System

Dietary fats are mostly triglycerides and are among the most important sources of nutrition in humans if taken appropriately. Many food sources contain various types of fatty acids, such as olive oil which is rich in monounsaturated fatty acids, animal products rich in saturated fats (but also with large proportions of monounsaturated and polyunsaturated fatty acids depending on the origin), plants rich in alpha linolenic acid, and nuts and seeds (such as walnuts and linseed), rich in omega 3 polyunsaturated fatty (105). Fatty acids are known to play diverse roles in immune cells (106, 107). Dietary fats are important for absorption of liposoluble vitamins A, D, E, and K (which are also involved in the immune system), as well as permeability and stability of immune cell membranes (108).

Short chain fatty acids (SCFAs), like acetate, propionate, and butyrate can be provided by many fermented foods made by bacterial fermentation such as cheese, butter, pickles, soy sauce, yogurt, and alcoholic beverages (109–113). Many studies have shown that SCFAs exert anti-inflammatory properties and present immunomodulatory potential *in vitro* (114, 115). SCFAs are able to regulate the activation, recruitment, and differentiation of immune cells, including neutrophils, dendritic cells (DCs), macrophages, and T lymphocytes (116). A study by Liu and colleagues showed that SCFAs not only reduced the production of pro-inflammatory factors, including TNF-α, IL-1β, IL-6, but also enhanced the production of the anti-inflammatory cytokine IL-10 (117).

Many studies have shown that palmitoleic acid (PA) (a monosaturated fatty acid belonging to the omega-7 group), also presents anti-inflammatory properties *in vitro* (118, 119). Dietary sources of palmitoleic acid include a variety of animal oils, vegetable oils, and marine oils. A recent study evidenced the role of the palmitoleic acid in decreasing pro-inflammatory cytokine expression in cultured macrophages characterized by a decrease in Th1 and Th17 response (120). Another important constituent of dietary fats is polyunsaturated fatty acids, which can be further subdivided into omega-3 and omega-6 fatty acids. Many studies using a variety of models show that a decrease in omega-6/omega-3 ratio has anti-inflammatory effects (121–125). A study using mice reported that the omega-3-derived lipid mediator protectin D1, significantly reduced influenza virus replication (126). Moreover, a randomized controlled trial showed that omega-3 supplements were able to lower inflammation in healthy middle-aged and older adults (124). The data showed that administration of 1,25 and 2.5 g/d of omega-3 decreased the IL-6 serum level by 10 and 12%, respectively (124). Another randomized control study showed that supplementation of omega 3 for 12 weeks reduced the production of IL-6, and lowered anxiety by 20%. These changes were accompanied by a decreasing ratio of omaga-6/omega-3 and consequent reductions in IL-6 and TNF-α production (127). Although the beneficial effect of omega-3 has been revealed by many studies, a caution with dose and the status of the body should be taken into consideration when this compound is taken. On the other hand, it has been shown that saturated and polyunsaturated omega-6 fatty acids present pro-inflammatory properties (107, 128). Furthermore, omega-6 fatty acids are precursors of potent lipid mediator signaling molecules, termed “eicosanoids,” which have important roles in the regulation of inflammation, and the eicosanoids derived from omega-6 also present pro-inflammatory properties (129). However, it should be mentioned that not all omega-6 have pro-inflammatory characteristics. Gamma-linolenic acid (GLA, 18:3n-6) is a precursor of eicosanoids, which is found in human milk and several botanical seed oils but is typically consumed as part of a dietary supplement. Several studies have shown that GLA can attenuate inflammatory responses (130, 131). Furthermore, it has been shown that polyunsaturated fatty acids are able to activate the peroxisome proliferator-activated receptors γ (PPAR-γ), thus decreasing the pro-inflammatory cytokines (132). For example, docosahexaenoic acid (DHA) and eicosapentaenoic acid (EPA) interact with PPAR-γ and leads to the inhibition of nuclear factor- κB (NF-κB), a key transcription factor of pro-inflammatory cytokine production (133). On the other hand, saturated fatty acids have been shown to trigger the secretion of pro-inflammatory mediators from various cell types, including macrophages (134, 135), adipocytes (136), astrocytes (137), and endothelial cells (138). An *in vitro* study also showed that the addition of palmitic acid to infected cells, by different strains of the influenza A virus, increased the cellular lipid content and thus increased the replication of the virus (139). This effect of palmitic acid has not been replicated for coronaviruses.

It has been reported that high-fat diets lead to increasing circulating pro-inflammatory cytokine and neutrophil levels, resulting in a poorer response to pandemic H1N1 influenza A virus (pH1N1) vaccination (140). In the same context, Milner and colleagues state that “Obesity has been identified as an independent risk factor for severe or fatal infection with 2009 pandemic H1N1 influenza (2009 pH1N1), but was not previously recognized for previous pandemic or seasonal influenza infections” (141). In this study, the authors showed that obese mice had elevated viral titers, greater lung inflammation, as well as increased inflammatory cytokine levels and damage, and more memory CD8+ CTL in the lung airways (141, 142). Moreover, HFD leading to obesity (animal model of obesity) can exacerbate inflammation or infection in the host, and consequently increase the mortality. This has been shown in obese mice infected with the influenza virus (143, 144), which was attributed to a delayed antibody response (141). In fact, infection of obese mice with the 2009 pandemic H1N1 influenza virus resulted in an elevation of pro- inflammatory cytokine concentrations in circulation, but a lower response of IFN-β and pro-inflammatory cytokine concentrations in the lungs, compared to lean mice (144). Similarly, another study with obese mice infected with the influenza virus showed that IFN-α and β were minimally expressed and there was a notable delay in expression of the pro-inflammatory cytokines IL-6 and TNF-α (143). The lower level of IFN-α and β leads to a less effective immune responses against viral agents (145). In this context, it has been shown that there is strong association between severity of COVID-19 disease and obesity (146). Thus, during the lockdown, individuals with a tendency for obesity and other metabolic disorders should avoid or reduce high fat meals since it has been shown that high fat diet have a detrimental role, downregulating ACE2 (147). Deregulation of ACE2 receptors in the airways allows easier entrance of the virus and leads to the increased angiotensin II release. In turn, this can cause vascular (endothelial) trauma and micro-thrombo-embolism in various organs, leading to multiple organ failure (82, 88).

Furthermore, high-fat dietary intake has been proven to be responsible for the alteration of microbial composition in the intestine by increasing the ratio of Firmicutes to Bacteroidetes leading to an increase in intestinal permeability. This may cause systemic inflammation thus affecting the immune system (140, 148). Trottier and colleagues observed induced inflammation in the immune system in mice that had been fed a high-fat diet. This was accompanied by a modest change in bone marrow composition and a slight increase in the percentage of lymphocytes (149).

**Figure 1:**
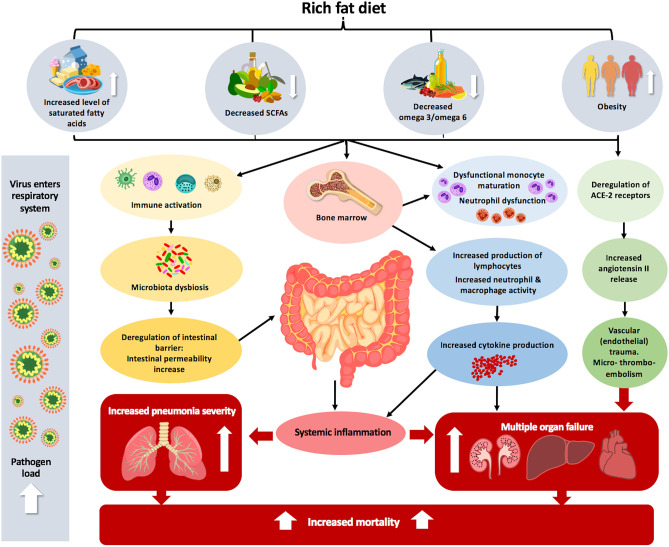
The effect of inadequate fat intake and obesity on the pathophysiology of COVID-19. Increased dietary level consumption of saturated fatty acids, decreased level of SCFAs and omega 3/omega 6 combined with obesity can lead to immune activation. This immune activation can alter microbial composition in the intestine, leading to dysbiosis, which consequently enhances systemic inflammation. The complexity of the intestinal microbiota is correlated with dysfunctional monocyte maturation and neutrophil dysfunction in the bone marrow. Obesity also leads to deregulation of ACE2 receptors in the lungs, which predisposes and makes entrance the virus easier and leads to increased angiotensin II release, which causes vascular (endothelial) trauma and micro-thrombo-embolism in various organs, leading to multiple organ failure. Altogether, these different factors that lead to the body being unbalanced can increase pneumonia severity and mortality, which is more acute in the case of lung viral infection.

In summary, the *in vitro* and *in vivo* studies using animal models indicate that fatty acids can directly modulate either negatively (high-fat diet, saturated and polyunsaturated omega-6 fatty acids) or positively (polyunsaturated omega-3, monounsaturated, and short-chain fatty acids) thereby affecting the immune response and influencing infection susceptibility (140) (Figure 1). However, a recent study in mice has shown that short term feeding (3–6 weeks) either with low-fat or high fat diets, rich with omega-3, omega-6 or monosaturated fatty acids, did not significantly influence the susceptibility of mice to viral infection, morbidity, viral titers in the lungs and liver, recovery time, or mortality (125).

#### Effect of Dietary Carbohydrates in the Immune System

Carbohydrates are nutrients found mainly in vegetables, fruits, and cereals and can be divided into simple sugars and oligo- or poly-saccharides. The recommended daily dietary allowance of carbohydrates is 130 g/day (150). Carbohydrates consumed as part of balanced diet are healthy but can be toxic if overconsumed. Carbohydrates are the most important fuel source and are necessary for the normal functioning of immune cells. Although an increase on lymphocytes during anaerobic glycolysis has been shown—which is an indicator of the increase of glucose as a fuel—during lymphocyte proliferation the use of this micronutrient as a source of energy decreases (151). Moreover, carbohydrates have an important impact on the immune system because of their ability to prevent the decrease of the number of cells conjoint to apoptosis (108). This fact is very important for COVID-19, because in severe cases there is an increase in apoptosis of lymphocytes.

On the other hand, a recent study showed that during times of stress (comparable to what many are facing during the COVID-19 pandemic) many people change their dietary behavior and tend to be drawn to unhealthy, high-sugar foods (152). A diet based on overconsumption of simple carbohydrates can lead to metabolic syndrome, an increase in abdominal fat, hyperglycemia, and type 2 diabetes, as well as dysregulation in the immune responses (151, 153). A recent paper by Goldberg and colleagues reported that feeding mice an energy dense, high-fat, low-carbohydrate ketogenic (keto) diet conferred protection in the context of a potentially lethal influenza infection. The authors identified that an energy dense, high-fat, low-carbohydrate ketogenic (keto) diet promoted the expansion of γδ T cells in the lung, leading to a conclusion that a keto diet may present a viable avenue toward preventing or alleviating influenza disease (154). Although this outcome was specific to mice and not to humans, it cannot be ignored that a keto diet may have beneficial effects for people with type 2 diabetes and other metabolic disorders (155–157) who have higher risk of complications if infected with SARS-CoV-2 (158). In this context, it has been revealed for example that 5.3–20% of COVID-19 patients in Wuhan had compromised innate immune responses because of diabetes (159, 160). A low carbohydrate diet has positive effects in people with type 2 diabetes (161) which may alleviate the severity of infection by SARS-CoV-2. Additionally, severe COVID-19 cases have exhibited increased catabolism, and therefore have increased energy requirements (162).

#### Effect of Dietary Proteins and Amino Acids on the Immune System

Proteins are considered the building blocks of life and their monomeric component, the amino acids, are considered key regulators of various pathological and physiological processes, including immune responses (163). The recommended daily dietary allowance of proteins is 19–56 g/day (150). It has been demonstrated that a deficiency of dietary protein and accompanying reduced concentrations of most amino acids in plasma, impairs the immune function and increases the susceptibility of humans to infectious diseases (164). A deficiency in protein intake is associated with the alteration of one of the first lines of defense against pathogens: the physical barrier. This deficiency is accompanied by thinner collagen and connective tissue, reducing the number of antibodies in the physical barrier, which results in a favorable environment for the aggressor (165). Moreover, the protein-energy malnutrition associated with chronic diseases has been recognized as a virulence factor for severe COVID-19 because it can deregulate immune cell activation leading to increasing inflammation in the lungs and longer viral persistence (133, 166). Moreover, it has been shown that COVID-19 patients require a diet rich in high energy nutrients (105–160 kj/kg/day or 25–40 kcal/kg/day) and proteins (167–170). In this context a protein intake >1 g/kg/day (up to 1.5–2 g/kg/day) has been proposed in COVID-19 patients that do not show any chronic renal insufficiency (22, 167).

There is increasing evidence on the important role of amino acids in the enhancement of the immune response, as well as in the reduction of an over-reaction, such as inflammation and autoimmunity (163). Thus, amino acids can regulate the activation of T and B lymphocytes, macrophages, NK cells, and the production of antibodies and cytokines (164, 171–173). Many amino acids like glutamine, arginine, tryptophan, cystine/cysteine, glutamate, histidine, and branched-chain amino acids are important for immune function (163). Some of them are discussed below.

##### Glutamine

This amino acid is the most abundant and versatile amino acid in the body. Research has shown that in health and disease, the rate of glutamine consumption by immune cells is similar to or greater than glucose consumption (174, 175). In fact, a decreasing level of glutamine in the plasma leads to: (1) a reduction in human B cell differentiation as well as a decrease in antibody production (176); (2) a suppression of T cell proliferation and decrease in IL-2; and (3) downregulation of major histocompatibility complex (MHC) class II antigen expression on human macrophages and inefficient phagocytosis (177).

##### Arginine

For many years, a diet rich in arginine, which is found abundantly in meats and nuts, often combined with other micro- and macronutrients, has been used as a mechanism to boost the immune system (178). It was reported that in experimental animals housed under stressful conditions, arginine supplementation was able to restore the reduced number of T cells to normal (163). Another study showed that L-arginine consumed through the diet can boost the activity of T cells. In fact, this study showed that an increase in the level of L-arginine reorganized the metabolism of the T cells, which made them more effective in fighting tumors and gave them a longer lifespan (179).

### The Role of Micronutrients in the Immune Function

Vitamins and other micronutrients are essential constituents of the human diet that have long been known to influence the immune system (165, 180). A deficiency in these micronutrients affects the innate and adaptive immune system response, leading to dysregulation of the balanced host response (181). Many studies have shown that vitamins A, B, C, D, E, minerals zinc, iron, magnesium, selenium, iodine, copper, and polyphenols among other micronutrients, have an important effect in supporting the immune system (182).

#### Vitamins

##### Vitamin C

Vitamin C is an essential micronutrient for humans that contributes to enhancing the immune response by supporting the innate and the adaptive immune system. The recommended daily dietary allowance of this micronutrient is 25–90 μg/day (150), and a deficiency in vitamin C deregulates the barrier function against pathogens, increases oxidative damage, and decreases phagocytosis (183, 184). In other words, vitamin C deficiency results in impaired immunity and increases the incidence and severity of pneumonia and other infections (182). Various studies showed that supplementation with a high dose of vitamin C stimulates phagocytic and T-lymphocytic activity in response to infection by increasing cytokine production and synthesis of immunoglobulins (182) and can help severely ill patients in intensive care to recover more quickly (182). A randomized, double-blind placebo-controlled trial in the UK showed that the administration of 200 mg/day of vitamin C to elderly patients with pneumonia reduced respiratory symptoms, mainly in patients with more acute respiratory infection (185). In a recent meta-analysis of nine randomized controlled trials, it has been shown that administration of a high dose of vitamin C (700–800 mg/day) against common cold virus infections lead to a reduction of the duration of infection and a shorter time of confinement (186). Although the used doses to treat pneumonia are higher than the RDA, a recent NIH document revealed that a diet with 1.5 g/kg body weight of vitamin C is safe and has no major adverse events (187). In fact the use of such high doses to treat infection, rather than the normal recommended doses, could be explained by the fact that during infection, the level of vitamin C decreases and the requirements of an infected person increases with the severity of the infection (188).

##### Vitamin D

Vitamin D is a fat-soluble vitamin that is naturally present in very few foods, but is available as a dietary supplement, and is produced by our body in response to sun exposure. The RDA of this micronutrient is 15–20 μg/day (150). Vitamin D has the capacity to maintain the structural and functional integrity of mucosal cells in innate barriers, such as the skin and the respiratory tract, which is very important during viral infection. In fact, this vitamin increases the tight junction protein expression, E-cadherin, and connection 43 in the gut, supporting the gut barrier (182). Moreover, vitamin D has various functional roles: it increases the differentiation of monocytes to macrophages (189) and it promotes the movement and phagocytic ability of macrophages (182). Also, this vitamin increases superoxide synthesis (182), reduces the expression of pro-inflammatory cytokines, and increases the expression of anti-inflammatory cytokines by macrophages (190, 191), all of which may enhance immune system reactivity. Vitamin D presents stimulatory effects in the innate immune system, promotes the production of Treg (182), and promotes antigen processing. A study conducted by Cannell and colleagues showed that calcitriol, an active form of vitamin D, was able to reduce the incidence of respiratory infections in children during epidemic influenza by restoring the immune function of macrophages (192). A recent review recommended that people at risk of influenza and/or COVID-19 take 250 μg/day of vitamin D3 for a few weeks followed by 125 μg/day (193). The same review stated that in order to treat infected people with COVID-19, higher vitamin D3 doses might be useful (193). A recent study with a group of 780 COVID-19 patients revealed that most positive patients with insufficient or deficient vitamin D status died (194). Moreover, Rhodes and colleagues highlighted that there is a low population mortality from COVID-19 in countries south of latitude 35 degrees North, supporting the hypothesis that vitamin D is a cofactor determining the severity of the infection and then the immune system response (195). Besides the various roles that vitamin D presents, this micronutrient could play a direct role in virus-receptor binding. In fact, it has been shown that vitamin D supplementation can reduce the number of virus particles that could attach to the ACE2 receptors and enter the cell by promoting the binding of the SARS-CoV-2 cell entry receptor ACE2 to AGTR1 (angiotensin II receptor type 1) (196). Altogether, although these data show that vitamin D can act at different stages of the immune response, administration of high doses of this vitamin as a therapy should be done under medical control mainly for individuals with diseases or disorders.

##### Vitamin A

Vitamin A is represented by many compounds, such as retinol, retinal, and retinoic acid, as well as various provitamin A carotenoids such as α- or β-carotene (197). Vitamin A, naturally found in foods from animal sources, including dairy products, fish, and meat, plays an important role in the regulation of innate and cell-mediated immunity and humoral antibody response (198, 199). The RDA of this micronutrient is 400–900 μg/day (150). A deficiency of vitamin A alters the integrity of mucosal epithelium, such as the eyes, gastrointestinal tract, and the respiratory system, which causes an increase in their susceptibility to many pathogens (199, 200). In fact, it has been shown that deficiency in vitamin A is associated with increased risk of infection (201) and is connected with an increased risk of developing respiratory inflammation and diseases in children (182). Moreover, vitamin A deficiency negatively affects neutrophil, macrophage, NK, and eosinophil cell functions (181, 182, 200, 202). Moreover, a deficiency in vitamin A may promote an excessive inflammatory response by increasing the production of IL-12, thus promoting T cell growth as well as the pro-inflammatory TNF-α, which induces inflammation and potentiates existing inflammatory conditions. Supplementation with vitamin A can reverse these effects (203, 204). Deficiency in this vitamin and its metabolites is also the cause of the alteration of Th1/Th2 balance by decreasing Th2 (200). Furthermore, a study revealed that persons with low vitamin A status showed an increased risk of lung dysfunction and respiratory disease (205). On the other hand, it has been shown that dietary supplementation with vitamin A in humans improves antibody titer response to various vaccines (204). Finally, Imad and colleagues suggested that vitamin A supplementation at 5–20 mg/day, may prevent morbidity and mortality in children from 6 months to 5 years of age (206).

Retinoic acid, the biologically active retinoid metabolite, has been shown to play an important role in the differentiation, maturation, and function of the innate immune system cells (207) and can also activate the NK cells (208). Different pre-clinical and clinical studies have shown that retinoids stimulate secretion and potentiate the effects of IFN-I, which represent a family of cytokines of the early innate immune response to viruses that are being tested against SARS-CoV-2 (209). In this context, it has been proposed that the key mechanism behind the relationship between retinoic acid and IFN-I, is the activation of the retinoic acid-induced gene I (RIG-I), which produces a pattern recognition receptor responsible for sensing RNA viruses, thus playing an important role in early innate anti-viral immune responses (209, 210).

Finally, some carotenoids serve as provitamins or precursors for vitamin A, and may thereby exert immune-modulating functions (196). In fact, it has been shown that carotenoids may regulate membrane fluidity and gap-junction communication (211). Another major factor that makes carotenoids important during the current pandemic is that this family of compounds has the potential to play antiviral roles (212). Furthermore, serum beta-carotene has been significantly associated with reduced risk of death from various diseases including respiratory diseases (213). In the same context, results from one study revealed that higher supplementation of some carotenoids (lutein/zeaxanthin) for people aged 65 years and over was associated with 23% lower respiratory mortality (214). Although the safe total carotenoid recommended intake range between 5.4 and 15.4 mg/day, supplementation with carotenoids should be taken with caution and high doses of β-carotene have been proposed to be prooxidant and toxic (215).

##### Vitamin E

Vitamin E, a known antioxidant, is found in many foods including vegetable oils, cereals, meat, poultry, eggs, fruits, vegetables, and wheat germ oil. The RDA of this micronutrient is 7–15 mg/day (150). Besides its antioxidant activity, vitamin E is able to optimize and enhance the immune response (181). A diet rich with vitamin E has been shown to protect cell membranes from damage caused by free radicals and support the integrity of epithelial barriers including those of the respiratory system (181). Supplementation with vitamin E, like vitamin A, promotes Th1 cytokine-mediated response accompanied by a decrease in Th2 response. Thus, this supplementation increases lymphocyte proliferation production of IL-2, NK cell cytotoxic activity, as well as the phagocytic activity by alveolar macrophages, which consequently cause an increase in resistance against infectious agents (182). Different studies have shown the effect of vitamin E in preventing infections such as the influenza virus (216). Moreover, a study conducted by Hemila showed that administration of 50 mg/day of vitamin E for 5–8 years may decrease the incidence of pneumonia by 69% in elderly males (217). Similarly, a randomized controlled trial with a total of 617 persons aged at least 65 years showed that a supplementation of 180 mg/day of vitamin E, which is much higher than the RDA, have an effect on lower respiratory tract infections (216).

##### Vitamin B

Vitamin B is a class of eight water-soluble vitamins that play important roles in cell metabolism. Many food sources are rich in vitamin B, including whole grains, legumes (beans and lentils), seeds and nuts, as well meat (especially liver). All three of these B vitamins are important because they are involved in the intestinal immune system, supporting the gut barrier, which is an important factor in maintaining an efficient immunity, as we will discuss later (218, 219).

##### Vitamin B6

Vitamin B6 is essential as a co-factor in nucleic acid, amino acid and protein biosynthesis, and therefore is important for proliferation, differentiation, and functioning of immune cells and synthesis of antibodies and cytokines (206, 220). An adequate diet rich in vitamin B should contain an average of 0.6–1.7 mg/day of vitamin B6 (150). Human studies demonstrate that vitamin B6 deficiency not only impairs lymphocyte maturation and growth, even with marginal deficiency, but also lowers the antibody responses as well as reduces responses to mitogens and T-cell activity (182). A deficiency in vitamin B6 also decreases the IL-2 production and NK cell activity and promotes Th2 cytokine-mediated activity, accompanied with a suppression of Th1 (182). It is important to emphasize that an adequate diet rich with vitamin B6 helps to restore cell-mediated immunity and has been shown to improve lymphocyte maturation and growth and increases the number of T-lymphocytes (182). Finally, Cheng and colleagues showed that a daily injection of 50 or 100 mg/day of vitamin B6 increased the immune responses in 51 subjects who stayed in an intensive care unit for over 14 days (221), suggesting that a higher dose than the one suggested by the RDA would have a beneficial effect, supporting the immune system of COVID-19 patients in an intensive care unit.

##### Vitamin B9 or Folate

Vitamin B9, similar to vitamins B6 and B12, plays an important role in protein synthesis. Therefore, a deficiency in vitamin B9 alters the immune system (165). An adequate diet rich in vitamin B should contain an average of 200–400 μg/day of vitamin B9 (150). A deficiency in vitamin B9 decreases the resistance to infections by inhibiting the proliferation and circulation of CD8+ CTL (221). Moreover, it has been shown that a deficiency in vitamin B9 impairs NK cytotoxicity (182). In this same context, a study including 60 healthy subjects aged over 70 years who received large intakes of vitamin B9 (supplementation of 400 mg/day), showed that the supplemented subjects reported an increase in NK cell cytotoxicity leading to fewer infections, suggesting that vitamin B9 supplementation increased innate immunity and provided protection against infections in elderly people (222).

##### Vitamin B12

Vitamin B12 is involved in carbon-1 metabolism and interacts with the folate metabolism (223). An adequate diet rich in vitamin B should contain an average of 1.2–2.4 μg/day of vitamin B12 (150). A deficiency in vitamin B12 causes suppression in NK cell activity, a decreased number of lymphocytes, a significant reduction in cells with a role in cell-mediated immunity, and changes in the proportions of CD8+ CTL and CD4+ Th, leading to abnormally high CD4+ Th/CD8+ CTL ratios (182, 219). A study of patients deficient in vitamin B12 showed that a supplementation with vitamin B12 reversed the effects that presented an abnormally high CD4+ Th/CD8+ CTL ratio and suppressed NK cell activity, indicating that this vitamin may act as a modulatory agent for cellular immunity, especially in relation to CD8+ CTL and NK cells (219). It has also been shown that a deficiency in vitamin B12 impairs the antibody response (181). Bunout and colleagues showed that a regular diet including 3.8 μg of vitamin B12 in elderly subjects (aged >70 years) over 4 months increases NK cell cytotoxic activity, leading to increased innate immunity in elderly people (222). Altogether, these studies state the importance of vitamin B12 in maintaining an adequate immune response, especially in older people (aged >65 years) who have low serum B12 concentrations (224).

##### Vitamin B2 (Riboflavin)

Vitamin B2 has a very important role in many energy-related enzymatic processes (196). The RDA of vitamin B2 is 0.6–1.3 mg/day (150). It has been suggested that vitamin B2 regulates fatty acid oxidation and therefore controls the differentiation and function of immune cells (225).

##### Vitamin B3 (Niacin)

Vitamin B3 is generally known as nicotinic acid and nicotinamide, which plays an important central role in aerobic respiration. The RDA of vitamin B3 is 8–16 mg/day (150). Vitamin B3 has been shown to modulate the host immune system by inducing the differentiation of Treg (226) and inhibiting the production of the pro-inflammatory cytokines IL-1, IL-6, and TNF-α by macrophages and monocytes (227).

##### Vitamin B5 (Pantothenic Acid)

Vitamin B5, like some of other B vitamins, is essential in the TCA cycle and fatty acid oxidation (228). The adequate intake (AI) of vitamin B5 is 3–5 mg/day (150). Vitamin B5, similar to vitamin B2, has been shown to be involved in the control of host immunity *via* energy generation by immune cells, which is very important in the case of COVID-19 patients (219).

##### Vitamin B7 (Biotin)

Vitamin B7 has a crucial role in nutrition and an important effect in immunometabolism. In fact, by being an essential cofactor for acetyl-CoA carboxylase and fatty acid synthase, this vitamin is used by the body to metabolize carbohydrates, fats, and amino acids (229). The AI of vitamin B7 is 12–30 μg/day for adults (150). Vitamin B7 deficiency induces Th1- and Th17-mediated pro-inflammatory responses in human CD4+ T lymphocytes (230). In the same context, a diet rich in vitamin B7 has anti-inflammatory effects and inhibits the activation of the transcription of NF-κB and thus inhibits the secretion of pro-inflammatory cytokines such as TNF-α, IL-1, IL-6, and IL-8 (231).

#### Minerals

##### Zinc

Whole grains, milk products, oysters, red meat, and poultry are good sources of zinc, and the RDA of this micronutrient is between 2 and 11 mg/day (150). Zinc is an essential micronutrient required for controlling key biological processes, and is involved in the regulation of both the innate and adaptive immune system (222). Zinc-deficient subjects may show severe disturbances in immune cell numbers and activities and may experience increased susceptibility to a variety of pathogens (222). Zinc is important for the structural and functional integrity of the skin and mucosal cells (189). Zinc-deficiency is manifested by an increased thymic atrophy, an imbalance in the Th1/Th2 ratio, characterized by a reduction in Th1 cell numbers, a decrease in lymphocyte proliferation and function, particularly T cells, and alteration in cytokine production—all of these contributing to greater oxidative stress and inflammation (181, 182). Zinc deficiency also impairs survival, proliferation, and maturation of monocytes, NK cells, T and B cells, phagocytosis by macrophages and neutrophils, as well as antibody responses to T cell-dependent antigens (181, 182). It has been shown that correction of zinc deficiency boosts the defense-related immune system, and reduces mortality from infectious diseases and viral infections (222, 232). From several controlled studies, it is clear that daily dietary supplementation of zinc for the elderly and children at high risk for zinc deficiency, is protective against infection and is associated with a decrease in mortality from infections in these populations (233–237). Furthermore, persons with a low zinc status have showed an increased risk of viral infections (238). A systematic review and a metanalysis study showed that zinc at doses of at least 75 mg/day is able to significantly reduce the duration of symptoms caused by viral infection on the upper respiratory tract but does not consistently improve the overall severity of symptoms (239).

##### Iron

This micronutrient is present in animal sources such as red meat and poultry, as well as in plants such as beans and lentils, cashews, spinach, and whole grains. It is important to note that the body absorbs two to three times more iron from animal sources than from plants. Iron is an essential micronutrient for the differentiation and growth of epithelial tissue as a first line of defense against pathogens (189). A diet rich in iron (10–18 mg/day) (150), or iron dietary supplementation, improves intracellular microbial killing and cellular immunity by forming toxic hydroxyl radicals, and is thus involved in the killing of pathogens by neutrophils and maintaining a certain level of lymphocyte bactericidal activity (189). Iron also has an important role in maintaining a certain level of IL-6 and IFN-γ production, as well as in the differentiation and the proliferation of T cells and in helping to regulate the ratio between CD4+ Th and CD8+ CTL (189). It has been shown that iron supplementation in children reduces the risk of respiratory tract infection (182). On the other hand, high doses of iron leads to increased viral mutations in the influenza virus genome resulting in a more virulent phenotype (240).

##### Magnesium

This micronutrient is present in greens, nuts, seeds, dry beans, whole grains, and low-fat dairy products. An adult diet containing 320–420 mg/day of magnesium can decrease oxidative stress by reducing the superoxide anion production, protecting the cells from oxidative damage (182). Magnesium also boosts the immune system by increasing NK-cell activity, regulating leukocyte activity and the ratio between CD4+ Th and CD8+ CTL, decreasing the levels of cytokines such as IL-6, and decreasing inflammation (182). Finally, it is important to note that magnesium is involved in antibody responses through antibody—particularly IgG—production, which is important in maintaining immune tolerance in order to distinguish between the “self” and the “non-self” (241).

##### Selenium

Among the nutrients implicated in viral infection, selenium is a nutritional antioxidant incorporated as a rare amino acid selenocysteine in selenoproteins (242). The RDA of this micronutrient is between 15 and 55 μg/day (150). Selenium plays an important role in antioxidant defense, by regulating reactive oxygen species (ROS) and redox status in tissues. Dietary selenium strongly influences inflammation and immune responses. Some *in vitro* studies on influenza showed that selenium deficiency resulted in reduced antioxidant activity of cells and an important increase in the pro-inflammatory cytokine IL-6, altering the response to influenza of epithelial cells (242). In addition, studies by Beck et al. (243, 244) showed that host selenium deficiency increased the virulence of RNA viruses such as coxsackievirus B3 and influenza A (242), while pointing at an interesting endemic disease in the northeast of China, where soil is selenium-deficient, namely Keshan disease. This disease is interesting to relate, as it is a seasonal cardiomyopathy for which the virus coxsackievirus B3 was identified as being a co-factor (243–245). Interestingly, when the population received a supplementation in selenium, the incidence of the disease decreased dramatically. In addition, selenium prevented mutations of the viral genomic RNA that lead to increased virulence and cardiac pathology (242). Finally, selenium was shown to be associated with a decrease in the occurrence of ventilator associated pneumonia in mechanically ventilated patients (246).

##### Iodine

It is well-known that a large number of people around the world do not consume enough iodine (247). However, deficiency is rare in developed countries because of iodized salt. The RDA of iodine is 150 μg/day for both males and females over 14 years old, while it increases to 220 μg/day during pregnancy and to 290 μg/day during breastfeeding (150). It has been shown that iodine presents a role in modulating the function of human immune cells and present some therapeutic effects in different pathologies (248, 249). A study showed that iodine is able to increase the movement of granulocytes into the area of inflammation and to improve their ability for phagocytosis, clearing infections (249). Furthermore, it has been reported that iodine has an indirect effect on the modulation of the immune system by modulating the thyroid hormone synthesis (248). The modulation of the thyroid hormones enhances NK cytotoxicity, the expression of cytokines as well as B cell differentiation and increases the frequency of T memory cells (248).

##### Copper

While enough dietary copper can be obtained from solids and water, it is important to mention the effect of copper deficiency, as it can occur in seriously ill individuals who require parenteral nutrition. The RDA of copper is 440–900 μg/day (150). Copper deficiency can also occur in older people as a result of malnutrition or malabsorption. Failure to correct this might lead to susceptibility to further infections by decreasing the number of circulatory blood cells (182, 250–252). Recent studies supported the role for Cu in the innate immune response against infections (250). Raha et al. hypothesized that copper supplementation can help to fight COVID19, especially in older people where a deficiency of Cu is a strong possibility (250). In fact, they suggested that a diet supplemented with Cu affects host immune function and metabolism of other micronutrients, prevents the severity of the viral infection and may protect people from COVID-19 (250). Finally, it is important to note that a wide array of lung infections can be accompanied by elevated copper levels (253) and that an accumulation of copper can also be toxic (254, 255).

#### Polyphenols

Polyphenols are produced in plants and can be classified into flavonoids, phenolic acids, polyphenolic amides, and other compounds (256). In addition to their well-established anti-inflammatory and anti-oxidant activities, studies have highlighted their antiviral potential. For example, antiviral properties of some polyphenols have been demonstrated against several viruses including Epstein-Barr, enterovirus, herpes simplex, and influenza (257). However, only a limited number of studies have investigated the role of polyphenols against coronaviruses directly (257). We will briefly cite the important polyphenols that have been tested in this regard. Ten polyphenolic compounds isolated from *Brussonetia* papyrifera proved effective against MERS/SARS-CoV proteases (258). Ethanolic extracts of Sambucus formosana proved effective against the human coronavirus strain HCoV-NL63 (259). Saikosaponin B2 has also shown good potency in this regard (258). Griffithsin is a polyphenol extracted from a red algae called Griffithsia genus and is one of the most promising inhibitors of MERS-CoV (258). By specifically binding to glycans of the CoV protein spikes, it can inhibit attachment of the virus to host cells, with high potency, making this polyphenol a good candidate for trials against SARS-CoV-2. Silvestrol is another polyphenol compound, extracted from *Aglaia* sp., that showed inhibitory properties against MERS-CoV (258).

Resveratrol (RSV) is probably the most promising polyphenol to test against SARS-CoV2. Indeed, it has been found to significantly inhibit MERS-CoV RNA replication *in vitro* on Vero E6 cells, *via* several mechanisms including inhibition of the virus protein expression, inhibition of the NFκB pathway and activation of the AMPK/Sirt1 axis in the host cell (257). RSV is found in mulberries, grapes, red wine, and peanuts, and was showed to possess—in addition to its antiviral properties—antioxidant, antitumoral effects, and scavenger of free radicals properties (260). A study tried to add RSV to the diet of piglets exposed to rotavirus and showed that RSV decreased TNF-α levels and diminished diarrhea in a resveratrol piglet diet (261). Another interesting study demonstrated the ability of RSV to counteract MERS-CoV infection by acting at different levels from reducing the cell death, inhibiting the viral replication, reducing the viral titer and inhibiting the expression of the nucleocapsid proteins, as well as inhibiting the apoptosis. This study demonstrates that RSV can be an adjunctive antiviral agent to consider in testing against SARS-CoV2. Finally a new clinical trial has been registered in the database clinicaltrial.gov to test the effect of resveratrol on COVID19 patients (NCT04400890) (262).

Although, data suggest that micronutrients play an important role in strengthening the immune system, it must be emphasized that the body requires optimal levels of micronutrients for effective immune function, with different requirements throughout every stage of life. For this reason, it is important to be aware that RDA for all nutrients is the *average* daily requirement necessary to avoid clinical or subclinical deficiency in the majority of people (97–98%) in a healthy general population (Table 3) (263). These RDA can be lower than effective therapeutic recommended doses needed to increase immune system responses in order to fight viral infections.

**Table 3:** Recommended dietary allowance.

**Macronutrients and micronutrients**	**Recommended dietary allowance**		
	**Children, M/F** **4–8 years** **9–13 years** **14–18 years**	**Adults, M/F** **19–50 years**	**Old age, M/F** **51–>70 years**
Fats, g/day	ND	ND	ND
Carbohydrates, g/day	130 130 130	130	130
Proteins, g/day	19 34 52	34/56	46/56
Vitamin C, mg/day	25 45 65/75	75/90	90/75
Vitamin D, μg/day	15	15	15/20
Vitamin A, μg/day	400 600 700/900	700/900	700/900
Vitamin E, mg/day	7 11 15	15	1.5/1.7
Vitamin B6, mg/day	0.6 1 1.2/1.3	1.3	1.5/1.7
Vitamin B12, μg/day	1.2 1.8 2.4	2.4	2.4
Vitamin B9, μg/day	200 300 400	300/400	400
Vitamin B2, mg/day	0.6 0.9 1.3	1.1/1.3	1.1/1.3
Vitamin B3, mg/day	8 12 16	14/16	14/16
Vitamin B5, mg/day	3^*^ 4^*^ 5^*^	5^*^	5^*^
Vitamin B5, μg/day	12^*^ 20^*^ 25^*^	30^*^	30^*^
Zinc, mg/day	5 8 11/9	8/11	8/11
Iron, mg/day	10 8 11/15	8/11	8
Magnesium, mg/day	130 240 360/410	310/420	420/320
Selenium, mg/day	30–40	55–70	55–70
Copper, mg/day	900–1,100	1,400–1,700	1,400–1,700
Iodine, mg/day	90–120	150	150

Except vitamin B5 and vitamin B7 where the values followed by an asterisk (^*^) represent the AIs, the values related to other micronutrients and micronutrients present the RDAs.

## Role of Probiotics, Diet and Fasting in Immune Function

### The Role of Probiotics in Immune Function

According to the FDA and the WHO, probiotics are defined as “live micro-organisms which can provide health benefits on the host when administered in adequate amounts” (264). Ever since probiotics were recognized for their beneficial effects on health, they have been used as potential dietary supplements (265). Probiotics or the gut bacteria produce various metabolites and co-metabolites as by-products of food metabolism (266). These molecules, produced by the gut microbiota, have the ability to cross the gut-blood barrier and affect the health through various mechanisms, such as energy supplementation for colonic epithelium and anti-inflammatory activity (267). One of the most important groups of metabolites produced by the gut microbiota through undigested fermented food are SCFAs (discussed in a previous section), such as acetic acid, butyric acid, propionic acid, that have been shown to have a beneficial effect by maintaining the integrity of the epithelial barrier, decreasing the “leaky gut,” and, as a consequence, triggering an inflammatory reaction and the modulation of oxidative stress and the immune response (268). In fact, probiotics are able to modulate the immune and the inflammatory response in the gut through their interaction with the gut mucosa and mucosal immune system, which host the largest part of the body's immune cells mainly within the gut-associated lymphoid tissue (263). Various studies have shown that probiotics are able to induce both: (1) the production of pro-inflammatory cytokines in order to facilitate the immune system against a further infection, and (2) the production of anti-inflammatory cytokines in order to have a balanced homeostasis by reducing an excessive inflammatory reaction induced by an infection (263). Moreover, probiotics' health benefits are not only limited to the intestinal tract, but also present modulatory effects in other locations of the mucosal system, such as the upper respiratory tract (269). In the same context, it has been shown that besides infecting the respiratory tract, SARS-CoV-2 can also infect the lower gastrointestinal tract, which is rich in ACE2 receptors (270).

Probiotics can have an effect on both the innate immune system and the adaptive immune system. Some probiotics achieve this beneficial effect by acting on the mucosal immune system, in particular DCs and NK cells (271). As an example, it has been shown that administration of *lactobacilli* to mice can enhance the immune function in mice by increasing NK cell activity and phagocytic activity of macrophages (272), as well as enhance the phagocytic capacity of peritoneal leukocytes (273), increase the expression of DC-maturation markers, and enhance lymphocyte proliferation (274). Consistent with studies using animal models, human studies also showed that probiotic use could have a positive effect on the immune system. Healthy, older individuals receiving *Lactobacillus rhamnosus* HN001 or *Bifidobacterium lactis* HN019 in a milk-based diet showed increases in their peripheral blood proportion of NK cells and their tumoricidal activity, as well as increases in phagocytic activity (275). Another study showed that a daily ingestion of fermented milk containing *Lactobacillus casei* DN114001 improved innate-defense capacity in 45 healthy, middle-aged people (aged 51–58 years) by increasing the oxidative burst capacity of monocytes as well as NK cells' tumoricidal activity (276).

There is also evidence that supplementation with probiotics has beneficial effects on the adaptive immune system by modulating the functions of both T and B cells while preventing an autoimmune inflammatory response (263). The effects of probiotics on T cells varies widely depending on the strain, going from promoting the production of Th1 (IFN-γ, IL-2, IL-12, TNF-α), Th17 (IL-17, IL-22), and Treg (IL-10, TGF-β) cytokines, to the inhibition of Th2 cytokines (IL-4) (208, 277). In animal studies, the administration of *Bifidobacterium bifidum* (5 × 10^8^ CFU/d) for 8 week for old mice, showed an enhancement of anti-oxidation activity in the thymus and spleen, alteration of gene expression, and improvement in immune function, leading to significantly increased cytokine IL-2 and IFN-γ levels but also decreased pro-inflammatory cytokines IL-6 and TNF-α concentrations (278). Mane and colleagues showed that the consumption of a skim milk rich with a mixture of *Lactobacillus plantarum* CECT 7315 and CECT 7316 for 12 weeks, enhanced systemic immunity in elderly subjects, manifested by fewer incidences of infection and mortality due to pneumonia, compared to those who received unenriched skim milk only (279). The study showed that the participants who consumed the skim milk enriched with probiotics had increased percentages of B cells, NK cells, CD4+, and CD8+ and that most of these changes lasted for another 12 weeks after stopping the consumption of the probiotics (279). Guillemard and colleagues conducted a double blind, controlled study, involving 1,072 volunteers (median age = 76.0 years) who were given a fermented dairy product containing the probiotic *Lactobacillus casei* DN-114001 (280). This study showed that supplementation with the fermented product was safe and was associated with a decrease in the duration of respiratory infections in comparison with the control group (280). A similar study showed that the consumption of yogurt fermented with *L. bulgaricus* OLL1073R-1, augmented NK cell activity and reduced the risk of infection and the risk of catching the common cold in elderly individuals (281). Altogether these studies suggest that the administration of probiotics can enhance the host's resistance against infection for older subjects and reduce the severity of viral infection in both the gastrointestinal tract and the respiratory tract.

Like probiotics, some selective prebiotics—which is defined as a substrate that is selectively utilized by host microorganisms conferring a health benefit—have also been reported to be beneficial for health. In this context, most of the studies considered that prebiotics have indirect effects on the immune system through changing the composition and population of gut microbiota (282). It has been shown that prebiotic compounds such as inulin, polydextrose, and maize fiber are able to improve the immune response, gut diversity, and digestion in humans—especially in elderly people (283, 284). In addition to the effects on the composition of the microbiota, prebiotics also produce notable shifts in the immune system by increasing the expression of anti-inflammatory cytokines, while reducing the expressions of pro-inflammatory cytokines (285, 286). Also, it is known now that prebiotics such as wheat bran, fructo-oligosachharides, and galactosachharides are known to increase butyrate levels thereby reducing inflammation and improving conditions in asthma and cystic fibrosis (287). It is to be noted that beneficial effects of the prebiotics are thought to be mediated mostly by increased production of SCFAs and strengthening of the gastrointestinal immune system. Overall, it is apparent that diet mediated modulation of gut microbiota, and to some extent even lung microbiota, can influence immunity and reduce the severity of viral infection in both the gastrointestinal tract and the respiratory tract (270, 287).

Taking into consideration that probiotics and prebiotics are generally safe, this microbiome therapy may improve and quicken the recovery of elderly patients and immune-compromised COVID19 patients. We suggest that probiotics/prebiotics that have been shown to have antiviral and respiratory benefits can be used as part of the actual therapies used to reduce infection with SARS- CoV-2. Nutritional recommendations could include a combinations of pre and probiotics (symbiotic), such as fructo-oligosaccharides and galactosaccharides, and various lactobacilli strains to improve gut dysbiosis, thereby improving the overall immune response (270, 288).

### Diet and Fasting

The health effects of various forms of fasting have been studied for decades and the database clinicaltrials.gov currently has 1,901 trials registered under the MeSH term “fasting” for a large array of diseases and disorders. Water fasting (which restricts everything except water), intermittent 16 h fasting, the fasting mimicking diet (FMD), and religious “Ramadan” fasting are the most common types of fasting under study. In particular, it is important to highlight the concurrent COVID19 pandemic with this year's “Ramadan” fasting. This is important because Islam has 1.8 billion adherents, the majority of whom were fasting during the pandemic. As this situation is highly unusual, many questions were raised as to whether fasting during the pandemic is safe or not. This situation has led physicians and scientists to consider the risks and benefits of fasting for their patients during the pandemic. This exceptional situation shows promise in providing data for observational clinical studies which will be shown progressively in future scientific literature (289–291). For these reasons, we will briefly review the risks and benefits of fasting during the COVID-19 pandemic.

A study in this regard conducted by Develioglu and colleagues revealed that lymphocyte numbers increased significantly, that serum IgG and salivary IgA decreased and that there were no changes in serum IgM (292). In fact, some evidence suggests that “Ramadan” fasting can actually change the functions of the immune system (291, 293). Other studies have shown the beneficial effect of intermittent, prolonged fasting during the month of Ramadan and how this could affect the inflammatory state (293–296). An investigation of 50 healthy volunteers who practiced “Ramadan” fasting was conducted 1 week before “Ramadan” fasting, at the end of the third week of “Ramadan,” and 1 month after the cessation of “Ramadan” (293). In this study, the authors showed that intermittent Ramadan fasting for a month, attenuated pro-inflammatory cytokines (IL-1β, IL-6) and decreased the number of lymphocytes, neutrophils, and monocytes in circulation as well as decreased the abdominal fat in healthy subjects (293). Similarly, another study on fasting for 1 month examined the effect of this prolonged intermittent fasting on serum cytokines levels in healthy and obese individuals (295). This study showed that the levels of different inflammatory biomarkers, including serum white blood cells (WBCs), IL-2, IL-8, and TNF-α, were significantly lower in both the control group and the obese group in comparison to pre-Ramadan values (295). Although these two studies showed that immune cells decreased during Ramadan but remained within the reference ranges, much more data are needed on this topic.

A recent study revealed that fasting can be quite safe for normal healthy individuals and can lead to “some beneficial changes in some inflammatory markers, as well as metabolic measurements” (297). Results showed decreased levels of pro-inflammatory chemokines GRO (growth-regulated oncogene)-alpha (Gro-α), IP-10, and stromal cell-derived factor 1 (SDF-1) in comparison with cytokine and chemokine profiles of COVID-19 patients that show marked elevation (298).

Furthermore, another study demonstrated that prolonged intermittent fasting has some positive effects on the inflammatory status (296). This study showed not only that the level of IL-6 decreased during fasting but the data also showed increases in circulating levels of vitamin B12 and folate, which have been previously found to be beneficial in supporting the immune system against viral infection. Another study showed that Ramadan fasting does not alter oxidative stress parameters or biochemical markers of cellular damage in healthy subjects. Although this study revealed a decrease in the level of carotenoids, which has previously been shown to exert immune-modulating functions (196), a slight reduction in lipid peroxidative damage in erythrocytes and no changes in retinol, vitamin E, and C have been observed (299). In fact, oxidative stress has been shown to be implicated on viral pathogenesis and infections (300, 301) and reducing lipid peroxidative damage in erythrocytes may reduce the consequences of viral infection.

It has been shown that fasting can decrease immunosenescence, extend life expectancy (302), reduce markers of oxidative stress and inflammation, and improve lung function, as well as to alleviate or reverse autoimmune disorders (303–305). The other studies on Ramadan fasting also showed reduced immune cell numbers, even though some found no changes (301). Only one study (292) on a small number of male subjects showed increased lymphocyte numbers.

Although some of the discussed results may support the hypothesis that fasting during the pandemic lockdown might not have a negative effect and might actually support the immune system response in case of an infection by SARS-CoV2, much more data are needed on this topic. One recent review and systematic analysis on the effects of Ramadan fasting on immunity by Adawi et al. (306) showed that the effects were diverse, and that the study samples were small, thus, a definite conclusion cannot be made.

## Conclusion

Nutrition and diet are able to promote the functioning of the immune system as a preventive measure by reducing both inflammation and oxidative stress that might be caused by various factors. Deficiencies in some micronutrients can increase inflammation and the risk of infection (196). Several of the micronutrients discussed in this review, can interact with transcription factors to regulate the expression of receptors used by viruses such as ACE2 (196). In addition, nutrition and diets modulate the gut microbiota, which can affect gut permeability and inflammatory status.

**Figure 2:**
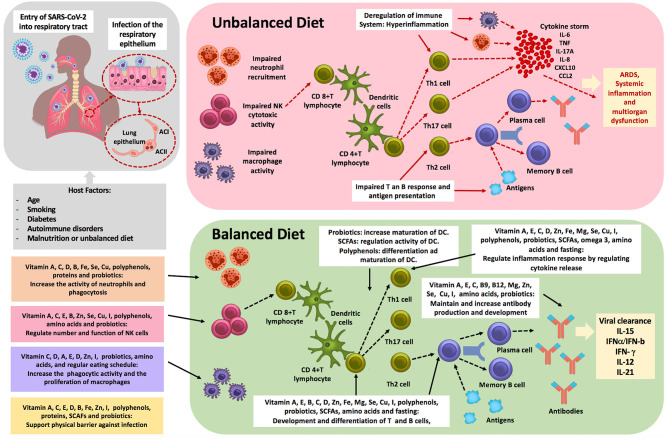
Important role of nutrition in strengthening the immune system in regard to the fight against SARS-CoV-2 infection. Red box: The effect of an unbalanced diet on the immune system response. Different host factors including age, smoking, diabetes, autoimmune disorders, malnutrition, or an unbalanced diet may affect the immune system response, leading to high levels of inflammation which explain the severe cases of COVID-19. In fact, in this case, invasion of the respiratory epithelium and other target cells by SARS-CoV-2 involves T-lymphocytes infection and apoptosis, leading to their decreased number and activity, and the consecutive impaired activation of B cells and the production and secretion of antibodies. This leads to the compensatory increased neutrophil and macrophage activity, their accumulation in the lungs and hyper-secretion of cytokines, in order to re-activate the adaptive immune system. The viral clearance is delayed and prolonged infection causes a decrease in ACE2 receptors, leading to over-activity of renin-angiotensin II system (RAS), which causes endothelial dysfunction and thrombosis. This could lead to a cytokine storm, accompanied by Respiratory Distress Syndrome (ARDS) and multiorgan dysfunction—characteristics of severe cases of COVID-19. Green box: The effect of a balanced diet on the immune system response. Vitamins A, C, D, B, E, iron, magnesium, zinc, copper, iodine, selenium, proteins, SCFAs, omega-3, a low-carb diet, polyphenols, probiotics, and a balanced diet were shown to directly support the body's natural defense system by enhancing the different levels of immunity and, therefore, might participate in the development of a strong immune system, which may help the body's immune system fight any viral infection and promote virus clearance.

It is essential that probiotics and necessary nutrients such as vitamins—which affect the immune system—are not neglected before and during infection. Vitamins A, C, D, B, E, iron, magnesium, zinc, copper, selenium, iodine, proteins, SCFAs, omega-3, a low fat diet, and polyphenols were shown to directly support the body's natural defense system by enhancing the different levels of immunity and therefore might promote virus clearance (Figure 2). It follows that infected patients who already have nutritional deficiencies or excess may have an inadequate inflammatory reaction causing more severe negative clinical outcomes.

Future clinical studies should not neglect the potential of minerals, vitamins, polyphenols, and probiotics in modulating the immune response (307). Moreover, close monitoring of micronutrient levels during treatment of COVID19 patients would contribute to a great advance in understanding the role of nutrition in treatment of COVID19.

## Author Contributions

AC conceived the study, researched, and wrote the manuscript. CM helped in editing the text and sketched the figures. GB and DZ helped in writing part of the manuscript and editing the text. All authors contributed to the proofreading of the manuscript and approved the final version of the manuscript.

## Conflict of Interest

The authors declare that the research was conducted in the absence of any commercial or financial relationships that could be construed as a potential conflict of interest.
